# Effect of Er Microalloying and Zn/Mg Ratio on Dry Sliding Wear Properties of Al-Zn-Mg Alloy

**DOI:** 10.3390/ma18153541

**Published:** 2025-07-29

**Authors:** Hanyu Chen, Xiaolan Wu, Xuxu Ding, Shengping Wen, Liang Hong, Kunyuan Gao, Wu Wei, Li Rong, Hui Huang, Zuoren Nie

**Affiliations:** 1 State Key Laboratory of Materials Low-Carbon Recycling, Beijing University of Technology, Beijing 100124, China; hanyu-chen@emails.bjut.edu.cn (H.C.); muselingxue@163.com (X.D.); wensp@bjut.edu.cn (S.W.); hongliang0422@163.com (L.H.); gaokunyuan@bjut.edu.cn (K.G.); weiwu@bjut.edu.cn (W.W.); rongli@bjut.edu.cn (L.R.); huanghui@bjut.edu.cn (H.H.); zrnie@bjut.edu.cn (Z.N.); 2 Weiqiao Lightweight Research Center at Soochow, Suzhou 215001, China

**Keywords:** Al-Zn-Mg alloy, Er-microalloying, microstructure, wear resistance

## Abstract

In this study, dry sliding wear tests were carried out on Er, Zr-microalloyed Al-Zn-Mg alloys with different Zn/Mg ratios under 30–70 N loads. The effects of the Zn/Mg content ratio and Er microalloying on the friction coefficient, wear volume loss, worn surface, and wear debris during the friction process of Al-Zn-Mg alloys were analyzed. At the load of 30 N, abrasive wear, fatigue wear, and adhesive wear were synergistically involved. At a load of 50 N, the abrasive wear dominated, accompanied by fatigue wear and adhesive wear. At a load of 70 N, the primary wear mechanisms transitioned to abrasive wear and fatigue wear, with additional adhesive wear and oxidative wear observed. Reducing the Zn/Mg ratio mitigated wear volume across all tested loads. For the Al4.5Zn1.5Mg alloy, Er microalloying significantly reduced wear volume under moderate-to-low loads (30 N, 50 N).

## 1. Introduction

High-strength Al-Zn-Mg-Cu alloys (7xxx series) are characterized by low density, high specific strength, and excellent hot workability, and have been widely used in key fields such as aerospace, aviation, rail transit, and energy [1,2,3,4]. With the expanding application scope of Al-Zn-Mg alloys, synergistic enhancement of strength and wear resistance has emerged as a pivotal challenge in overcoming current service limitations of lightweight engineering materials [5,6,7,8,9,10,11,12,13]. The wear resistance of materials after abrasion typically exhibits a strong correlation with subsurface hardness [14,15,16]. Research by Chegini [9] and Elhefnawey [10] has shown that equal-channel angular extrusion (ECAP) reduces the grain size of 7075 and 7050 alloys by approximately 100 times, with the wear rates decreasing by about 35% and 38%, respectively. The wear mechanisms transitioned from a combination of adhesive wear, oxidative wear, and delamination wear to predominantly abrasive and oxidative wear. Yin [16] investigated the effects of cryogenic-temperature extrusion machining (CT-EM) on the microstructural evolution and wear resistance of 7050 aluminum alloy. Compared to room-temperature extrusion machining (RT-EM), the CT-EM process suppressed dynamic recovery, further refined the average grain size, and improved wear resistance. Nevertheless, current research on wear resistance of 7xxx series alloys predominantly focuses on modifying alloys through various processing techniques. There remains a significant gap in exploring composition design strategies, such as regulating alloy composition and introducing microalloying elements, to improve wear resistance.

Adjusting alloy composition through micro-alloying to improve mechanical properties, thereby enhancing wear resistance, has emerged as a promising approach [17,18,19,20,21,22]. Studies have demonstrated that the addition of trace Er and Zr elements can effectively refine the microstructure and enhance the mechanical properties of aluminum alloys [23,24]. Nano-scale precipitates significantly inhibit dislocation motion and grain boundary migration through Zener pinning effects, thereby achieving substantial grain refinement and improving overall mechanical performance [25,26,27,28,29]. Quantitative analysis by Li [30] confirmed that adding 0.15 wt.% Er reduces the average grain diameter of as-cast alloy by 50%, decreases recrystallized grain fraction by 20% in the solution-treated state, and elevates the contribution of grain boundary strengthening by 89%. The comprehensive evolution of the microstructure indicates that Er and Zr microalloying holds promise for positively influencing the wear resistance of aluminum alloys. Wu [22] further reported that the addition of Er and Zr increases the hardness of Al-Sn alloy by 6%, improves strength by 7%, reduces volume wear loss by 22%, and significantly enhances wear resistance. In Al-Zn-Mg alloys, the mechanisms by which Er and Zr composite microalloying enhances mechanical properties have been extensively investigated. However, given their comprehensive effects on alloy strength and plasticity, research gaps persist regarding the influence of Er and Zr on wear resistance and wear mechanisms, which require further investigation.

In this study, to investigate the effects of alloy composition on the tribological behavior of Al-Zn-Mg alloys, a series of Al-Zn-Mg alloys with varying Zn/Mg ratios and Er contents was fabricated, and dry sliding wear tests were conducted. The influence of Er/Zr micro-alloying and the Zn/Mg ratio on the frictional behavior and wear resistance of the alloys was systematically investigated based on the wear coefficients and wear volume losses. The worn surfaces, tribo-layers, and debris of the alloys were characterized by scanning electron microscopy (SEM) and energy-dispersive spectroscopy (EDS) to elucidate the wear mechanisms under different compositional and loading conditions.

## 2. Experimental

### Experimental Material

A comprehensive analysis of the literature concerning the Zn/Mg ratio and tribological performance of 7xxx series aluminum alloys [31,32,33,34,35] reveals that industrial Al–Zn–Mg alloys typically contain a total (Zn + Mg) content of 5–8 wt.% and exhibit Zn/Mg ratios ranging from 1.5 to 3.5. Therefore, it is determined that the total content of Zn + Mg in the alloy in this study is 6%, the Zn/Mg ratio is designed to be 2:1 and 3:1, and the alloy group with a 0.1 wt.% Er element is designed to investigate the effect of Er on the wear performance of Al-Zn-Mg alloy.

High-purity aluminum, pure zinc, pure magnesium, Al-6 wt.% Er, and Al-10 wt.% Zr master alloys were used as raw materials for melting at 800 °C. The average compositions of ingots determined by X-ray fluorescence (XRF) sampling using PANALYTICAL (Almelo, the Netherlands) AXIOSMAX are listed in Table 1. To obtain finely dispersed Al_3_(Er_1−*x*_,Zr*_x_*) precipitates, a two-stage homogenization regime (350 °C/10 h + 475 °C/12 h) was implemented following references [36]. The homogenized ingots were hot-rolled at 450 °C to a deformation of 90%, followed by a solution treatment of the alloy at 470 °C/2 h and an isothermal aging treatment at 120 °C.

**Figure 1:**
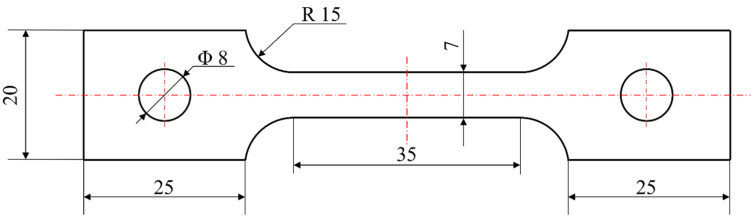
Dimensions of tensile specimens (Unit: mm).

The peak aging regime was determined as 120 °C/48 h through comparative analysis of age-hardening responses under varying holding durations. Room-temperature tensile tests were subsequently performed on peak-aged alloy specimens using an MTS System (Eden Prairie, NA, USA) MTS810 universal testing machine. The dimension of the tensile test specimen is shown in Figure 1. The tensile testing procedure strictly adhered to ASTM E8/E8M-21 standard [37] specifications. Based on the Archimedes principle, the density of the heat-treated alloy was measured by the Mettler (Zurich, Switzerland) ME204E balance with the components for density measurement.

**Figure 2:**
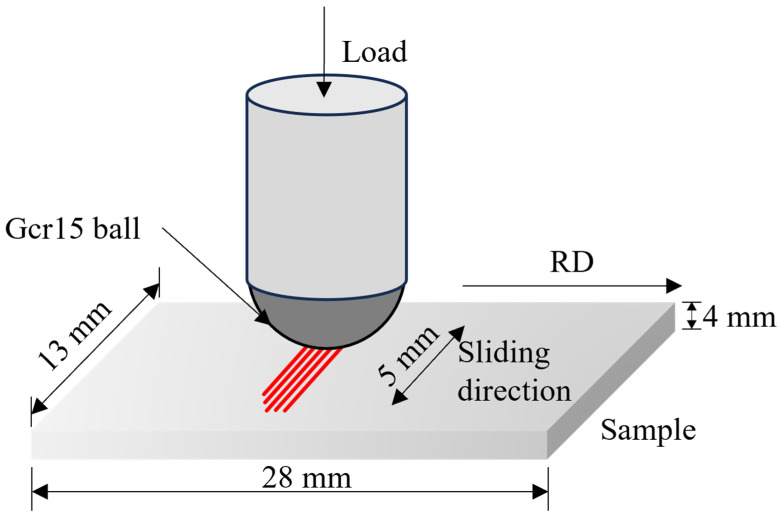
Schematic diagram of reciprocating dry sliding experiment.

The reciprocating dry sliding experiment of each group of alloys was carried out by using ZHONGKEKAIHUA (Lanzhou, China) CFT-1 material surface comprehensive tester. The worn surface was polished to Ra = 0.05 μm before the experiment. The schematic diagram of the dry sliding process is shown in Figure 2. In the experiment, the specimen size of the dry sliding wear test is 28 mm × 13 mm × 4 mm. The friction pair was a GCr15 steel ball with a diameter of 5 mm. Dry sliding is carried out perpendicular to the rolling direction of the specimen at room temperature. The load of the dry sliding wear test is 30 N, 50 N, and 70 N; the reciprocating stroke distance is 5 mm, the frequency is 300 r/min, the dry sliding time is 30 min, and the total sliding distance is 90 m. For each sample, two replicate experiments were conducted. Specimens were weighed using the Mettler ME204E balance before and after testing to determine the mass wear loss. Wear debris generated during the dry sliding process was collected using conductive adhesive. The worn surface, wear cross-sections, and debris morphology and microstructure were analyzed using an FEI (Hillsboro, OR, USA) Helios Nanolab 600i scanning electron microscope (SEM).

## 3. Results and Discussion

### 3.1. Mechanical Properties and Microstructure Analysis

**Figure 3:**
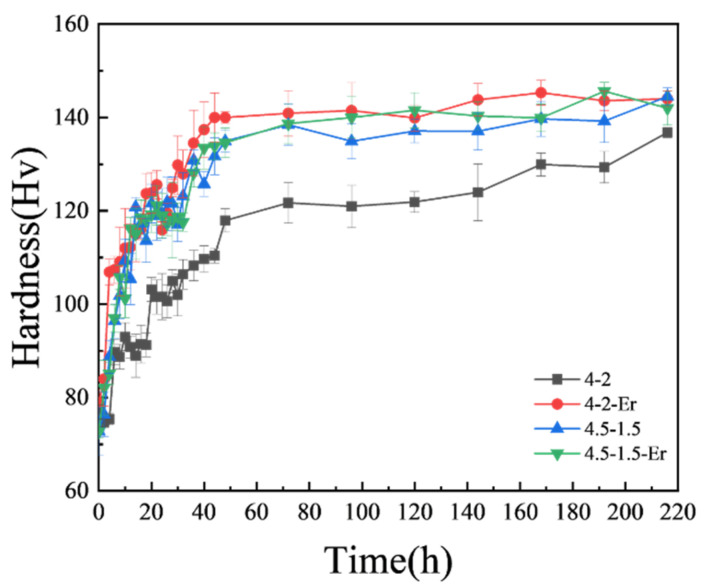
The 120 °C single-stage aging hardness curve.

**Figure 4:**
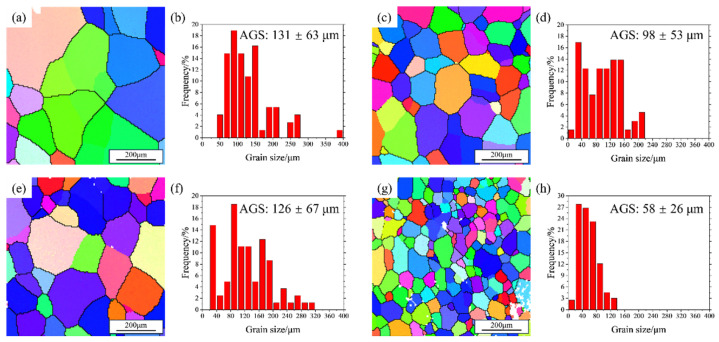
EBSD microstructure of solution-treated alloy: (**a**,**b**) Al4Zn2Mg; (**c**,**d**) Al4Zn2Mg0.1Er; (**e**,**f**) Al4.5Zn1.5Mg; (**g**,**h**) Al4.5Zn1.5Mg0.1Er.

The mechanical properties of the alloys in peak-aged condition are presented in Table 2. Within 48 h, the Al4Zn2Mg alloy exhibited the lowest strength and hardness among the four alloys, accompanied by the highest elongation of ~19%. As shown in Figure 3, the hardness of the Al4Zn2Mg alloy gradually increased from 118 HV (48 h) to about 136 HV (216 h) after aging at 120 °C, indicating that the supersaturated solid solution was not fully precipitated after aging for 48 h. The addition of the Er element enables microalloying with Er and Zr, leading to the formation of Al_3_(Er, Zr) precipitates, which act as nucleation sites to promote the precipitation of the primary aging strengthening phase in the Al-Zn-Mg alloy [38], thus improving the strength of the Al4Zn2Mg0.1Er alloy and reducing the elongation from 19% to 17%. By increasing the Zn/Mg ratio of the alloy, the content of the Zn element, which is easier to diffuse in the alloy, increases, thus accelerating the precipitation of the strengthening phase [31,39]. Consequently, the Al4.5Zn1.5Mg alloy exhibited higher strength but lower elongation compared to the Al4Zn2Mg alloy after 48 h of aging. Microstructural characterization via EBSD (Figure 4) demonstrated that Er addition refined grain size from 131 μm to 98 μm (by 25%) in the alloy with a Zn/Mg ratio of 1.7:1. A more pronounced grain refinement was observed in the alloy with a Zn/Mg ratio of 2.8:1, where grain size decreased from 126 μm to 58 μm (by 54%), indicating a composition-dependent grain refinement effect. This aligns with prior studies [29] confirming that nano-scale Al_3_(Er_1−*x*_, Zr*_x_*) precipitates formed in the matrix. These particles inhibit grain boundary migration and recrystallized grain growth through Zener pinning, thereby enhancing both strength and ductility via grain refinement.

### 3.2. Wear Rate

**Figure 5:**
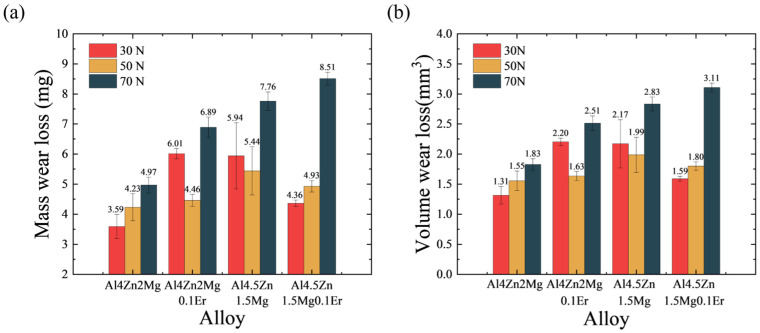
Comparison of mass wear loss (**a**) and volume wear loss (**b**) of alloy under different loads.

The volume wear loss and mass wear loss of specimens during dry sliding wear under various loads is presented in Figure 5 and Table 3. Alloys with a Zn/Mg ratio of 2.8:1 exhibited increased wear volume loss across all load conditions. Under moderate-to-low loads (30 N and 50 N), Er microalloying enhanced the elongation of the Al4.5Zn1.5Mg0.1Er alloy through grain refinement. This improvement enhanced the alloy’s plastic flow capability during wear, thereby reducing its volume wear loss. For the Al4Zn2Mg0.1Er alloy, the elongation improvement achieved via Er-induced grain refinement could not compensate for the reduction in elongation caused by Er-accelerated aging precipitation of η’ phase. Consequently, the volume wear loss of the Al4Zn2Mg0.1Er alloy increased compared to that of the Al4Zn2Mg alloy. Due to its higher Zn/Mg ratio, which promotes the aging precipitation of the η’ phase and consequently reduces elongation, the Al4.5Zn1.5Mg alloy exhibited a higher volume wear loss than the Al4Zn2Mg alloy. Simultaneously, the wear losses of all alloy compositions escalated progressively with increasing applied loads. At a load of 70 N, the alloys experienced significantly intensified wear compared to 30 N and 50 N conditions, accompanied by substantial increases in wear volume loss. This observation suggests a potential transition in wear mechanisms under high-load conditions.

### 3.3. Wear Coefficient

**Figure 6:**
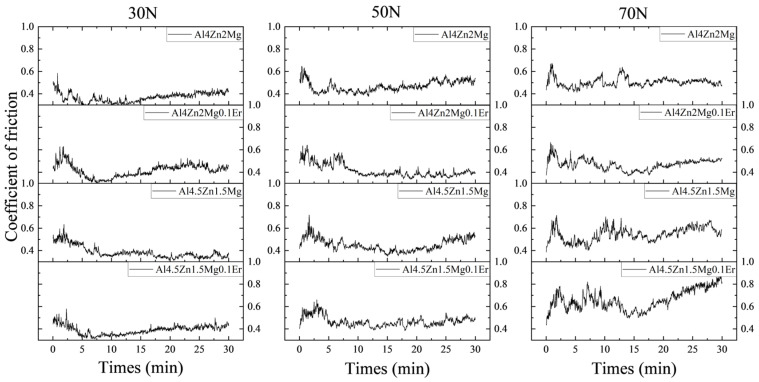
Comparison of the coefficient of friction of the alloy under different loads.

**Figure 7:**
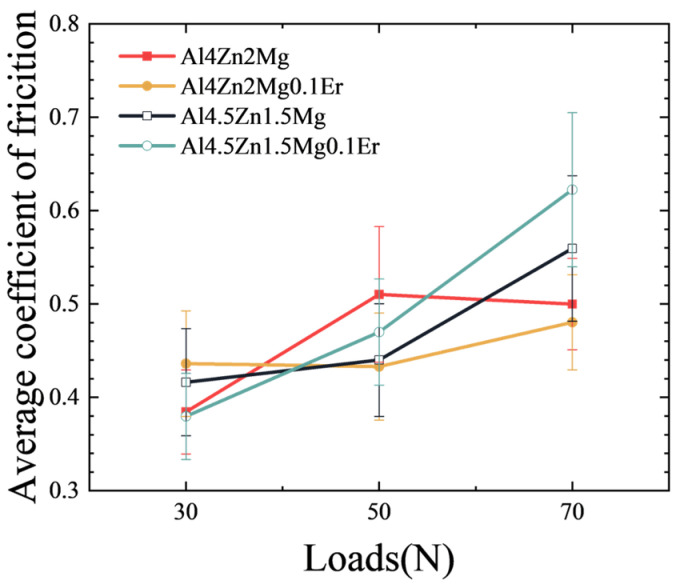
Variation in average coefficient of friction with load for the alloy.

Figure 6 illustrates the coefficient of friction (CoF) curves of the alloys with different compositions under experimental conditions. During the initial running-in stage of wear, significant fluctuations in the CoF curves were observed, characterized by one or more friction peaks. The limited asperity contact points and elevated surface roughness at this stage contributed to higher volume wear loss [40]. As surface asperities became flattened and roughness decreased with the formation of a stable mechanically mixed layer, CoF curve fluctuations diminished, marking the transition to steady-state wear. Both the fluctuation amplitude of CoF curves and the required running-in duration increased with rising applied loads. The running-in period extended from approximately 8 min at a load of 30 N to 15 min at a load of 70 N. The average friction coefficient during steady-state wear was calculated, with the computational results presented in Figure 7. Notably, the friction coefficient exhibited a marked elevation under high loads (50 N and 70 N). At the loads of 50 N and 70 N, alloys with a Zn/Mg ratio of 3:1 exhibited progressively higher friction coefficients.

### 3.4. Worn Surfaces

**Figure 8:**
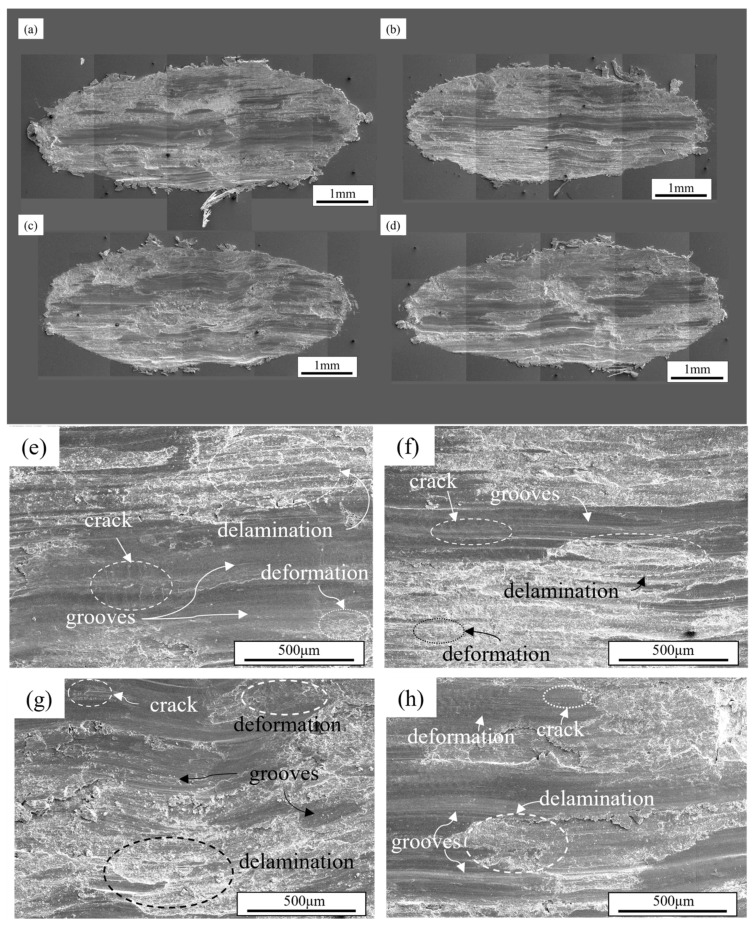
SEM morphologies of worn surface under 30 N load: (**a**,**e**) Al4Zn2Mg; (**b**,**f**) Al4Zn2Mg0.1Er; (**c**,**g**) Al4.5Zn1.5Mg; (**d**,**h**) Al4.5Zn1.5Mg0.1Er.

During the wear process, worn surfaces with distinct morphological characteristics were formed on material surfaces under different wear mechanisms. The worn surface of the alloy under a load of 30 N is shown in Figure 8. Figure 8a–d presents the overall view of the alloy worn surface, where a small amount of large material fragments that underwent plastic deformation during wear and were extruded to the edges of the worn surface can be observed. All worn surfaces exhibit noticeable surface delamination accompanied by numerous grooves parallel to the sliding direction, confirming the occurrence of significant abrasive wear [40,41]. Compared with the Al4Zn2Mg0.1Er alloy, the Al4Zn2Mg alloy exhibits a higher amount of clustered wear debris accumulated at the edge of the worn surface, indicating that Er microalloying enhances the alloy’s strength and improves its resistance to plowing by promoting the precipitation of strengthening phases and refining the grain size. The magnified views of worn surface (Figure 8e–h) demonstrate no significant differences among various compositions. Worn surface analysis revealed characteristic delamination from fatigue wear alongside adhesive wear features, including uneven delamination and rippling delamination, indicating a synergistic effect of three wear mechanisms—abrasive wear, adhesive wear, and fatigue wear—at the load of 30 N. At relatively low loads, the stress concentration at the asperity tips of the counterfaces remains moderate with shallow penetration depth, resulting in lower volumetric wear loss.

**Figure 9:**
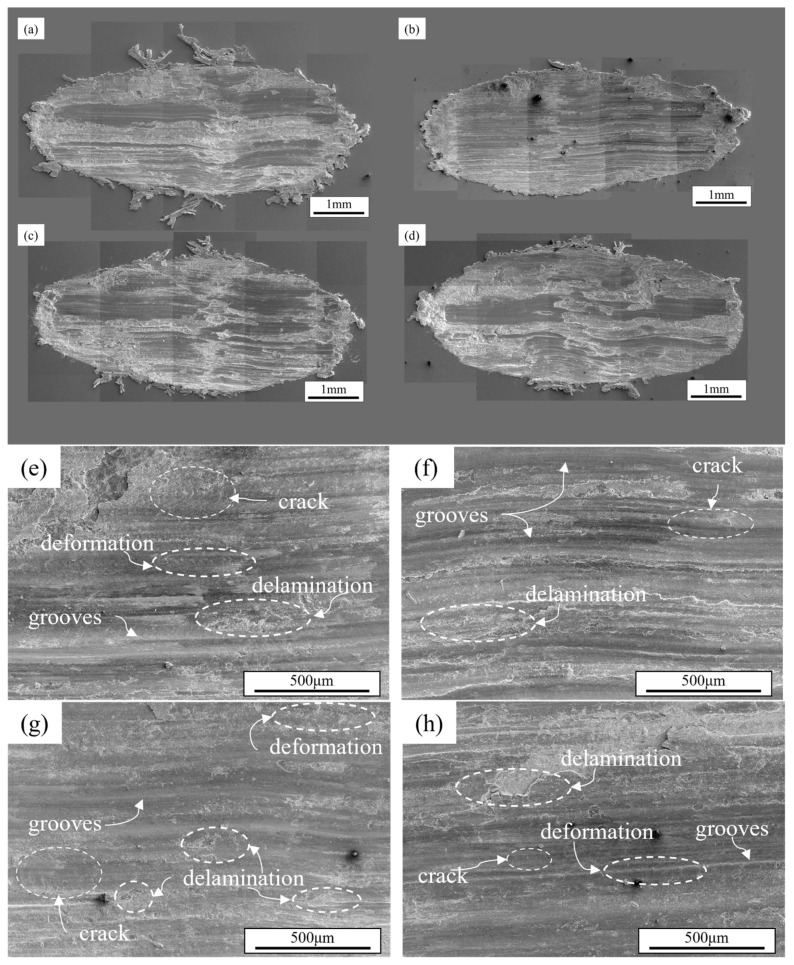
SEM morphologies of worn surface under 50 N load: (**a**,**e**) Al4Zn2Mg; (**b**,**f**) Al4Zn2Mg0.1Er; (**c**,**g**) Al4.5Zn1.5Mg; (**d**,**h**) Al4.5Zn1.5Mg0.1Er.

Figure 9a–d present the overall morphology of worn surface for various alloy compositions under a load of 50 N. Compared with Figure 8a–d, the plowing effect on alloy surfaces becomes more pronounced at 50 N, with increased accumulation of clustered wear debris at both ends of the worn surface and more material extruded through plastic deformation at the edges. When the load is increased to 50 N, enhanced deformation depth during friction generates greater volumes of plastically deformed microstructures, resulting in higher volumetric wear loss. The Al4Zn2Mg alloy, with an elongation of 19%, exhibits superior deformation capability compared to the other alloy compositions, and thus retains more extruded material at the edges of the worn surface. Distinct plowing grooves are observable in all alloy worn surfaces, with numerous grooves also visible in the magnified views shown in Figure 9e–h. Furthermore, the worn surfaces exhibited numerous parallel-aligned microcracks perpendicular to the sliding direction, delamination resulting from the coalescence of these microcracks, and plastic deformation zones induced during the wear process. The spalling areas characteristic of adhesive wear appear reduced compared to lower load conditions, suggesting that abrasive wear becomes more dominant at 50 N, while adhesive wear and fatigue wear play relatively minor roles. Er microalloying refined the grains of the Al4.5Zn1.5Mg0.1Er alloy, resulting in a marked improvement in elongation compared to the Al4.5Zn1.5Mg alloy. This grain refinement enhanced fatigue crack propagation resistance, thereby improving fatigue wear resistance and reducing volume wear loss [42,43]. Conversely, the Al4Zn2Mg alloy retains a coarser grain size. Nevertheless, its less pronounced aging precipitation process minimizes the detrimental effect of precipitation strengthening on ductility. Therefore, the alloy obtained the highest elongation and excellent crack propagation resistance, and obtained the lowest volume wear loss under each test wear load.

**Figure 10:**
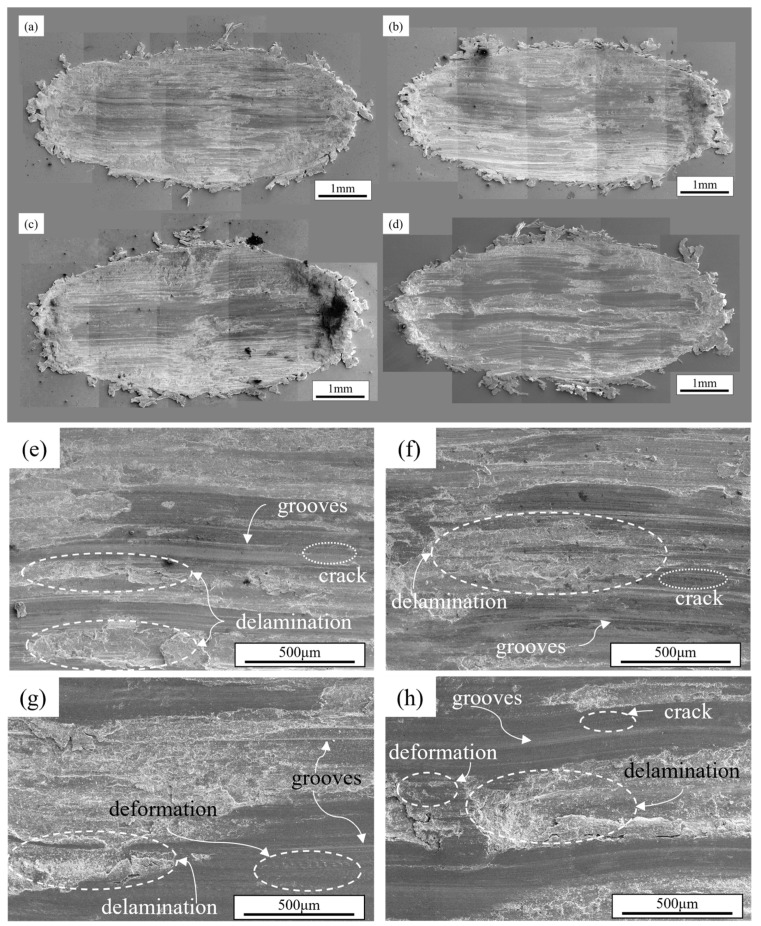
SEM morphologies of worn surface under 70 N load: (**a**,**e**) Al4Zn2Mg; (**b**,**f**) Al4Zn2Mg0.1Er; (**c**,**g**) Al4.5Zn1.5Mg; (**d**,**h**) Al4.5Zn1.5Mg0.1Er.

Figure 10 presents the worn surfaces of alloys under a load of 70 N. A substantial amount of material exhibiting severe plastic deformation characteristics has accumulated at the scar edges, showing significantly greater accumulation compared to that observed at 30 N and 50 N loads. Clustered wear debris accumulates at the ends of worn surfaces. Pronounced plastic deformation and spalling phenomena are visible on the worn surfaces. At a load of 70 N, prolonged dry sliding causes severe surface damage, resulting in considerably higher wear loss. Within localized worn surface, severe delamination and spalling exposed substantial areas of the substrate and subsurface. Discernible adhesive signatures are present, accompanied by numerous wide/deep plowing grooves and dense microcracks. These features indicate severe abrasive wear, fatigue wear, and concurrent adhesive wear during friction. Under elevated loads, accelerated propagation of microcracks within the alloy occurred concurrently with enhanced adhesion between the alloy surface and the friction pair, leading to pronounced delamination and elevated volume wear loss. Comparative analysis of the wear scars of the different alloy compositions reveals that as the degree of surface spalling of the alloy increases, so does the wear volume of the alloy.

### 3.5. Wear Debris

**Figure 11:**
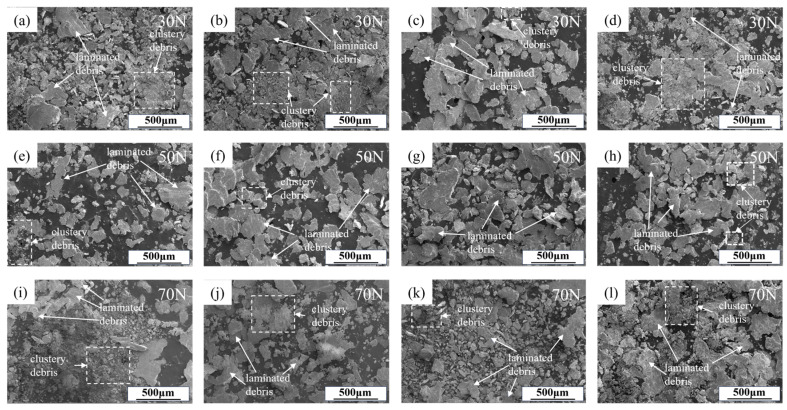
Wear debris of alloy under different load: (**a**,**e**,**i**) Al4Zn2Mg; (**b**,**f**,**j**) Al4Zn2Mg0.1Er; (**c**,**g**,**k**) Al4.5Zn1.5Mg; (**d**,**h**,**l**) Al4.5Zn1.5Mg0.1Er.

Figure 11 presents the morphology of wear debris generated from alloys under various loading conditions. As direct products of reciprocating dry friction interactions between alloys and friction pairs, the debris reflects their mutual interaction mechanisms. During sliding, fine wear debris generated by the ploughing action of counterface asperities agglomerated into clustered debris under compressive stress [44,45]. Under cyclic compressive stress from the friction pair, microcracks initiated at stress-concentrated regions in the material’s surface layer propagate and progressively coalesce, ultimately inducing surface delamination that generates the laminated debris [46,47]. Notably, certain debris particles exhibit significantly larger dimensions with observable plastic deformation characteristics on their surfaces, which are presumably generated through fatigue spalling of the surface layer caused by cyclic stresses during friction.

At the load of 30 N, all four alloy compositions exhibited a considerable amount of laminated wear debris. With the exception of the Al4.5Zn1.5Mg alloy demonstrating only 13% elongation, clustered wear debris was observed in the other three alloys. Notably, the Al4Zn2Mg alloy with 19% elongation showed the highest proportion of clustered wear debris. These observations suggest that both Er microalloying and reduction in the Zn/Mg ratio effectively enhance the alloy’s elongation, consequently promoting the formation of more clustered wear debris during the wear process.

At a load of 50 N, the wear debris of all alloy compositions was predominantly composed of the laminated debris, accompanied by a small quantity of large-sized debris extruded from the worn surface edges. Under elevated loads, increased surface stress accelerated the nucleation and propagation of microcracks, thus generating greater quantities of laminated debris. Comparative analysis of the wear debris reveals that the alloy with the lowest elongation (Al4.5Zn1.5Mg) yields debris composed almost exclusively of laminated debris, with clustered debris being virtually absent. In contrast, the other three alloys exhibit both delaminated debris and a measurable proportion of clustered debris, indicating that even modest improvements in elongation can slightly suppress fatigue wear.

At a load of 70 N, the wear debris of three alloys (Al4Zn2Mg, Al4Zn2Mg0.1Er, and Al4.5Zn1.5Mg0.1Er) with elongations exceeding 17% exhibited substantial amounts of both clustered and laminated debris, accompanied by minor quantities of larger debris generated through fatigue wear. In the Al4.5Zn1.5Mg alloy with comparatively lower elongation, partial clustered wear debris formation is also detected, demonstrating uniform proportional distribution and dimensional characteristics across debris types. Compositional adjustments that enhance alloy plasticity reduce the contribution of fatigue wear mechanisms, shifting the dominant wear mode toward abrasive wear.

### 3.6. Cross-Sectional Surface Analysis

**Figure 12:**
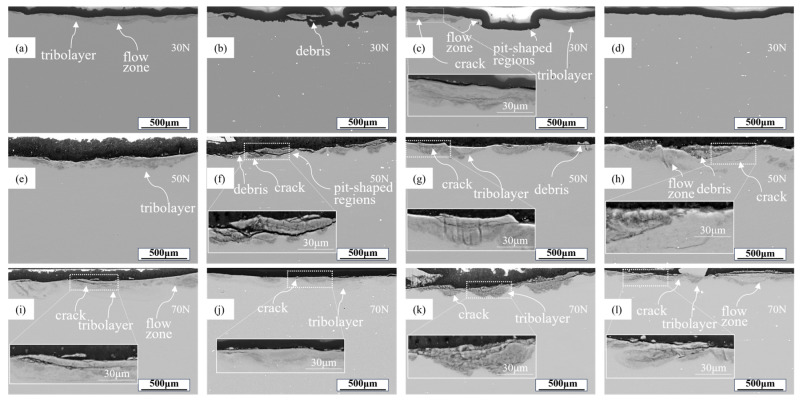
Subsurface BSE diagram of worn surface: (**a**,**e**,**i**) Al4Zn2Mg; (**b**,**f**,**j**) Al4Zn2Mg0.1Er; (**c**,**g**,**k**) Al4.5Zn1.5Mg; (**d**,**h**,**l**) Al4.5Zn1.5Mg0.1Er.

Figure 12 presents the backscattered electron (BSE) images of the subsurface wear layers of the alloys under different loads. The wear subsurface layers in 7xxx series alloys typically consist of composite layers formed by newly formed oxide layers and sheared matrix material, which combine with plastic deformation layers from the alloy matrix and are separated by wavy interfaces [48].

At the load of 30 N, the worn surfaces and subsurface layers exhibited material flow induced by plastic deformation during friction, accompanied by spalling caused by deformation fatigue. The tribo-layer contained large cracks and voids, with adjacent areas populated by fine debris particles.

At a load of 50 N, the alloy underwent more pronounced plastic deformation, evidenced by expanded flow zones [49], while the tribo-layer thickness increased significantly compared to 30 N conditions. Interactions between asperities of the friction pair and the metal surface triggered localized spalling, forming pit-shaped regions in the subsurface layer [12].

At a load of 70 N, rapid dynamic evolution of the friction layer prevented substantial thickness growth, while crack density increased sharply along with organized fragmentation, consistent with the extensive microcracks shown in Figure 10. Er micro-alloying increased primary phases in the matrix, which acted as crack initiation sites to promote crack proliferation and propagation, exacerbating wear loss under high load. Comparative analysis of tribolayers across alloys reveals that the Al4Zn2Mg alloy exhibits optimal material flow continuity and the least severe spalling. Among all alloy compositions, the Al4Zn2Mg alloy demonstrates the highest elongation, suggesting more homogeneous deformation during friction that consequently enhances its wear resistance. Integrated interpretation of elongation data (Figure 2) and wear loss comparisons (Figure 5) suggests that plasticity serves as the primary determinant of tribological performance in Al-Zn-Mg alloys.

**Figure 13:**
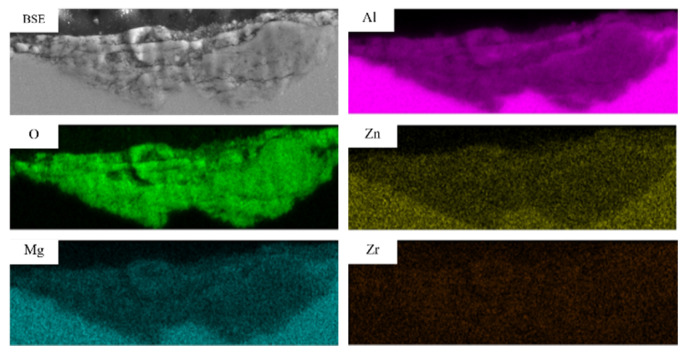
EDS analysis of wear subsurface of Al4.5Zn1.5Mg alloy under 70N load.

At a load of 70 N, the EDS analysis results of the subsurface layer of Al4.5Zn1.5Mg alloy (Figure 13) revealed significant oxygen enrichment, confirming the occurrence of oxidative wear during the process [50,51]. Notably, oxide clusters exhibited pronounced accumulation at tribo-layer defect [52], where fragmented hard oxide particles—generated from the fracture of oxide layers—acted as abrasive media. These particles induced three-body wear and intensified oxidative wear, synergistically accelerating material degradation.

### 3.7. Specific Wear Rate Discussion

**Figure 14:**
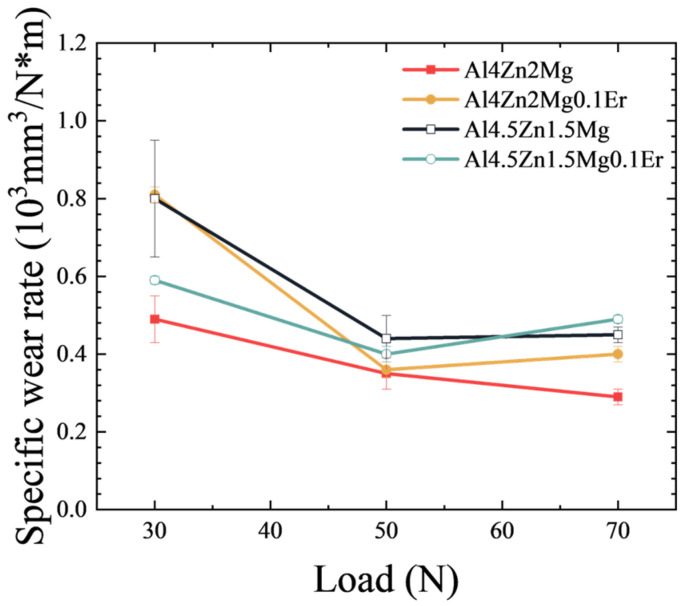
Variation in specific wear rate with load.

Archard’s theory points out that the surface condition of the alloy is related to the evolution of the specific wear rate with the load. Based on the alloy specific wear rate formula [53,54]:

(1) $$W=\frac{V}{NS}$$

where *V* represents the wear volume loss of the alloy, *N* denotes the experimental load, and *S* is the sliding distance. The specific wear rate can be calculated for different loads, with the results shown in Figure 14. The Al4Zn2Mg alloy exhibits the lowest volume wear rate under all conditions. Increasing the load from 30 N to 50 N notably reduced the specific wear rate of the alloy, whereas further elevation to 70 N resulted in statistically unchanged specific wear rates. At a load of 30 N, all four alloys exhibited relatively high specific wear rates. Coupled with extensive delamination regions observed on worn surfaces (Figure 8), this suggests instability of the tribolayer at this load, where spalling readily occurred, driving elevated specific wear rates. When the load is increased to 50 N, more subsurface grains are deformed, which promotes the formation of a stabilized tribolayer resistant to delamination [55,56,57]. As evidenced by reduced spalling areas in Figure 9 (vs. Figure 8) and increased tribolayer thickness in Figure 12e–h (vs. Figure 12a–d), this enhanced the wear resistance. Upon further increasing the load to 70 N, no significant change in SWR is observed. Comparable tribolayer thicknesses between Figure 12i–l and Figure 12e–h indicate similar stability of tribolayers under loads of 50 N and 70 N. However, accumulated microcracks within worn surface (Figure 10) and tribolayers (Figure 12i–l) imply that elevated loads promote crack propagation, ultimately increasing volume wear loss.

Based on the aforementioned observations, at a load of 30 N, the alloy surfaces developed numerous grooves and delamination zones, accompanied by substantial laminated debris generated through abrasive wear-induced crack propagation, along with minor amounts of clustered wear debris formed during the plowing process. These features collectively reflect the occurrence of multi-mechanism wear involving abrasive wear, fatigue wear, and adhesive wear. Pronounced delamination on worn surfaces indicates tribolayer instability under these conditions, leading to elevated specific wear rates during sliding (Figure 14). Nevertheless, the relatively low load resulted in low volume wear loss (Figure 5b).

At a load of 50 N, pronounced ploughing grooves accompanied by microcracks and coalescence-induced delamination were observed on alloy surfaces. The laminated debris is dominant in the wear debris, and the clustered debris appears in the alloy with high elongation, indicating that predominant abrasive wear with concurrent fatigue wear at this load. Enhancing alloy elongation modestly improves fatigue resistance, thereby yielding a slight reduction in volume wear loss. Under higher loading conditions, deeper plastic deformation zones develop in the subsurface region, facilitating the formation of stabilized tribolayers. This tribolayer effectively minimized direct contact between the base material and the friction pair, thereby enhancing tribolayer stability, reducing the extent of worn surface delamination, and consequently decreasing the specific wear rate of the material (Figure 14). Nevertheless, at an elevated load of 50 N, the reduction in specific wear rate proved insufficient to compensate for the increased load severity, ultimately resulting in higher volume wear loss than at a load of 30 N (Figure 5b).

When the load increases to 70 N, severe delamination spalling and broad/deep grooves manifest on alloy surfaces, accompanied by substantial clustered wear debris and laminated debris. This indicates the occurrence of compound wear mechanisms combining abrasive wear and fatigue wear. The thickness of the tribolayer remains essentially unchanged, indicating that it still possesses high stability; consequently, the specific wear rate (Figure 14) does not differ significantly from that measured at 50 N. Nevertheless, the higher applied load (70 N, compared to 30 N and 50 N) still leads to an increase in volume wear loss (Figure 5b).

## 4. Conclusions

The conclusions of this study on the reciprocating dry friction experiments of multi-component Al-Zn-Mg alloy sheets under different loads are as follows:

1. Er micro-alloying induced pronounced grain refinement and further shortened the peak aging duration for alloys with a Zn/Mg ratio of 1.7:1. Compared with the Al4.5Zn1.5Mg alloy, Er micro-alloying marginally enhances the ductility of the Al4.5Zn1.5Mg0.1Er alloy.
2. Reducing the Zn/Mg ratio mitigated wear volume across all tested loads. For the Al4.5Zn1.5Mg alloy, Er microalloying significantly reduced wear volume under moderate-to-low loads (30 N, 50 N)
3. Er microalloying did not alter the wear mechanisms. At the load of 30 N, the wear mechanism of the Al-Zn-Mg alloy is a combined action of abrasive wear, fatigue wear, and adhesive wear. At a load of 50 N, abrasive wear becomes the dominant mechanism, accompanied by fatigue wear and partial adhesive wear. At a load of 70 N, the primary wear mechanisms of the alloy are abrasive wear and fatigue wear, with adhesive wear being of secondary importance.
4. Due to the formation of the stabilized tribolayers, the specific wear rate decreased with load elevated from 30 to 50 N. When the load increased from 50 N to 70 N, the alloy’s tribolayer thickness remained stable, maintaining specific wear rate. Notwithstanding, crack proliferation within the tribolayer at a load of 70 N caused aggravated volume wear loss.

## Figures and Tables

**Table 1:** Chemical composition of alloys (wt.%).

Alloy	Zn	Mg	Er	Zr	Al	Zn/Mg Ratio
Al4Zn2Mg	3.61	2.17	-	0.09	Bal.	1.66
Al4Zn2Mg0.1Er	3.80	2.13	0.08	0.10	Bal.	1.78
Al4.5Zn1.5Mg	4.43	1.58	-	0.10	Bal.	2.80
Al4.5Zn1.5Mg0.1Er	4.47	1.60	0.09	0.09	Bal.	2.79

**Table 2:** Mechanical properties of alloy under 120 °C peak aging.

Alloy	YS (MPa)	UTS (MPa)	Elongation (%)	Hardness (HV_0.3_)	Density (g/cm^3^)
Al4Zn2Mg	298 ± 10	355 ± 6	18.7 ± 2.1	118 ± 3	2.721
Al4Zn2Mg0.1Er	332 ± 11	379 ± 8	17.2 ± 1.2	140 ± 1	2.730
Al4.5Zn1.5Mg	407 ± 3	444 ± 3	12.8 ± 0.6	135 ± 2	2.739
Al4.5Zn1.5Mg0.1Er	405 ± 1	443 ± 2	16.7 ± 0.9	135 ± 3	2.740

**Table 3:** The mass wear loss and volume wear loss of alloy under different loads.

Alloy	Mass Wear Loss (mg)			Volume Wear Loss (mm^3^)		
	30 N	50 N	70 N	30 N	50 N	70 N
Al4Zn2Mg	3.59 ± 0.40	4.23 ± 0.45	4.97 ± 0.26	1.31 ± 0.15	1.56 ± 0.16	1.83 ± 0.10
Al4Zn2Mg0.1Er	6.01 ± 0.17	4.46 ± 0.20	6.86 ± 0.34	2.20 ± 0.06	1.63 ± 0.07	2.51 ± 0.12
Al4.5Zn1.5Mg	5.94 ± 1.10	5.44 ± 0.80	7.76 ± 0.31	2.17 ± 0.40	1.99 ± 0.29	2.83 ± 0.11
Al4.5Zn1.5Mg0.1Er	4.36 ± 0.11	4.93 ± 0.19	8.51 ± 0.21	1.59 ± 0.04	1.80 ± 0.07	3.11 ± 0.08

## Data Availability

The original contributions presented in this study are included in the article. Further inquiries can be directed to the corresponding author.
